# Interventions in health organisations to reduce the impact of adverse events in second and third victims

**DOI:** 10.1186/s12913-015-0994-x

**Published:** 2015-08-22

**Authors:** José Joaquín Mira, Susana Lorenzo, Irene Carrillo, Lena Ferrús, Pastora Pérez-Pérez, Fuencisla Iglesias, Carmen Silvestre, Guadalupe Olivera, Elena Zavala, Roberto Nuño-Solinís, José Ángel Maderuelo-Fernández, Julián Vitaller, Pilar Astier, on behalf of the Research Group on Second and Third Victims

**Affiliations:** Departamento de Salud Alicante-Sant Joan, Alicante, Spain; Universidad Miguel Hernández, Altamira Building, Avd Universidad s/n, 03202 Elche, Spain; Hospital Universitario Fundación Alcorcón, Madrid, Spain; Consorcio Sanitario Integral, L’Hospitalet de Llobregat, Barcelona, Spain; Observatorio para la Seguridad del Paciente, Agencia de Calidad Sanitaria de Andalucía, Sevilla, Spain; Servicio de Salud de Castilla La Mancha (SESCAM), Toledo, Spain; Atención Primaria Comarca Donostia, Donostia, Spain; Servicio Madrileño de Salud, Madrid, Spain; Hospital Universitario Donostia, Donostia, Spain; Instituto Vasco de Innovación Sanitaria (O+berri), Barakaldo, Spain; Gerencia de Atención Primaria de Salamanca. Castilla y León Health Service (SACYL), Salamanca, Spain; Inspección Médica, Elche, Spain; Medicina de Familia y Comunitaria, Centro de Salud Caspe, Sector Alcañiz, Servicio Aragonés de Salud (SALUD), Zaragoza, Spain

## Abstract

**Background:**

Adverse events (AE) are also the cause of suffering in health professionals involved. This study was designed to identify and analyse organization-level strategies adopted in both primary care and hospitals in Spain to address the impact of serious AE on second and third victims.

**Methods:**

A cross-sectional study was conducted in healthcare organizations assessing: safety culture; health organization crisis management plans for serious AE; actions planned to ensure transparency in communication with patients (and relatives) who experience an AE; support for second victims; and protective measures to safeguard the institution’s reputation (the third victim).

**Results:**

A total of 406 managers and patient safety coordinators replied to the survey. Deficient provision of support for second victims was acknowledged by 71 and 61 % of the participants from hospitals and primary care respectively; these respondents reported there was no support protocol for second victims in place in their organizations. Regarding third victim initiatives, 35 % of hospital and 43 % of primary care professionals indicated no crisis management plan for serious AE existed in their organization, and in the case of primary care, there was no crisis committee in 34 % of cases. The degree of implementation of second and third victim support interventions was perceived to be greater in hospitals (mean 14.1, SD 3.5) than in primary care (mean 11.8, SD 3.1) (*p* < 0.001).

**Conclusions:**

Many Spanish health organizations do not have a second and third victim support or a crisis management plan in place to respond to serious AEs.

**Electronic supplementary material:**

The online version of this article (doi:10.1186/s12913-015-0994-x) contains supplementary material, which is available to authorized users.

## Background

Adverse events (AE) are the cause of harm and suffering in patients and may also markedly affect the work, family and personal life of health professionals involved [1], second victims, as well as damaging the reputation of affected health organisations (third victims), by undermining people’s trust in these institutions [2, 3]. Between 28 and 57 % of physicians [4–7] (79–89 % in the case of residents [8, 9]) recognise having being involved in medical error with serious consequences for one or more patients at some point in their career, while 90 % [6] believe that in their hospital there is insufficient help and support for professionals following an AE. In Spain, extrapolating from national AE data in hospitals [10] and primary care [11], it has been estimated [2] that 15 % of healthcare professionals are involved in this type of event per year.

Professional and personal consequences affecting second victims, together with different supportive interventions attempted to address the associated stress, have been reviewed in various studies published between 2008 and 2015 [3, 12–15]. White et al. [16] identified different strategies for managers of healthcare organisations to deal with the issue of second victims. Personal consequences for second victims are typically anxiety, negative emotional symptoms and loss of confidence in their professional skills and performance. We now know that these second victims change the way they interact with patients in the aftermath of serious AE and that they become insecure about their practice [3, 6, 12, 14]. Strategies to tackle stress, such as Critical Incident Stress Management and the BICEPS (Brevity, Immediacy, Centrality, Expectancy, Proximity and Simplicity) used by the military for the management of stress in the workplace, have been taken as a reference for approaches to the situation of second victims [13, 16, 17]. However further research is needed to determine the effectiveness of this approaches in reducing second victim symptoms.

The research into the consequences for third victims is very scarce [15, 18], nevertheless it has been suggested the implementation of a crisis management plan and associated measures could limit potential damage to an organisation’s reputation [2]. In relation to this, health organisations and their management teams could implement many types of interventions to prevent or minimise the negative impact of AE on second and third victims. To our knowledge, studies conducted to date have mostly focused on the hospital setting, and none have investigated AE impact on primary care clinicians.

The objective of this study was to identify and analyse the approaches taken in primary care and hospitals in Spain to address the impact of serious AE on second and third victims.

## Methods

We conducted a cross-sectional study. The reference population was composed of the managers and patient safety coordinators of hospitals and primary care health districts of the regional health services in Andalusia, Aragón, Castilla La Mancha, Castilla y León, Catalonia, Valencia, Madrid and the Basque Country, 8 of the 17 autonomous communities (regions) of Spain. These eight regions included in the study provide health cover to 76 % of the Spanish population and the corresponding autonomous regions accounted for 78 % of the GDP in 2013 [19]. The hospitals invited to participate in the study handled 75 % of all hospital admissions and the health districts 75 % and primary care consultations in Spain during 2012, according to the latest data published by the Spanish Ministry of Health [20, 21].

A web based questionnaire was designed and sent out to the following professionals: 326 hospital and primary care centre heads of medical staff or managers (*N* = 199 and *N* = 127, respectively) and 307 patient safety coordinators in hospitals (*N* = 129) and primary care health districts (*N* = 178). This study was conducted between February and April 2014.

An adverse event (AE) was understood as an incident that results in non-intentional harm to a patient, as an unexpected clinical result of healthcare, but which may or may not be related to a clinical error, as defined by as the World Health Organization [22]. A serious AE was defined as one which caused considerable harm (requiring of new treatment or prolonged hospitalisation) or irreparable damage (erroneous surgery, permanent disability or death). A second victim was defined as a health care provider involved in an unanticipated adverse patient event, medical error, and/or a patient related-injury who become victimized in the sense that the provider is traumatized by the event [15, 17]. A third victim was defined as a health organisation, which can also suffer a potential reputation loss from the patient safety incident [15, 18].

Invitations to participate were sent to a manager in each of the public hospitals and primary care health districts and to all the patient safety coordinators in these eight regional health services. Candidates were invited to participate in the study and requested to give consent, explaining their voluntary participation and the anonymity of their responses as well as the rationale and objectives of the study.

### The questionnaire

The questionnaire used for the survey explored five intervention areas: safety culture; health organisation crisis management plans for serious AE; measures to ensure transparency in communication with patients (and relatives) who experience an AE; care and support for second victims; and actions to protect the reputation of the health organisation (the third victim). It was developed by consensus among the research team on the basis of reviews, namely those of Brandom et al. [13], White et al. [16] and Denham [18], reviewing recommendations for managers, and interventions for second victims proposed and/or implemented by a wide range of organisations. For this study, we considered approaches from (Table 1): Massachusetts General Hospital (checklist for coaches supporting colleagues after AE), the Institute for Healthcare Improvement, the Agency for Healthcare Research and Quality, Medically Induced Trauma Support Services, Missouri Hospital, Washington University Hospital in St Louis, the National Quality Forum (Care of the Caregiver, an endorsed safe practice), Johns Hopkins Hospital and the Institute for Patient Safety Excellence at the University of Illinois at Chicago (the Seven Pillars process), Kaiser Permanente (support for the patient care team), Physicians Insurance (the Adverse Event Response Team [AVERT] Program) and Brigham and Women’s Hospital, Boston (peer support program), in the USA; the Royal College of Physicians, in the UK; and Miguel Hernández University (checklist developed by the CALITÉ group), in Spain.

**Table 1:** Recommendations, sources of information and initiatives reviewed

From the USA
Institute for Healthcare Improvement
http://www.ihi.org/resources/Pages/Publications/SupportingInvolvedHealthCareProfessionalsSecondVictims.aspx
Agency for Healthcare Research and Quality
http://www.ahrq.gov/news/newsroom/commentaries/second-victim-syndrome.html
Medically Induced Trauma Support Services
http://www.mitss.org
Missouri Hospital
http://www.muhealth.org/secondvictim
Institute for Patient Safety Excellence at the University of Illinois at Chicago
http://www.cade.uic.edu/sphapps/SPHpub/R/New_Map/Abstract_Summary_t4.asp?pnbr='2010-03738'
Physicians Insurance (the Adverse Event Response Team [AVERT] Program)
http://mikegreenstein.com/media/docs/Physicians-Insurance-Annual-Report-2010.pdf
Kaiser Permanente (support for the patient care team)
http://www.ihi.org/resources/Documents/KaiserPermanenteImplementationGuidelinesCommunicatingUnanticipatedOutcomesOct02.pdf
Johns Hopkins Hospital
http://m.hopkinsmedicine.org/news/publications/dome/november_2010/the_second_victims
Brigham and Women’s Hospital, Boston (peer support program)
http://www.brighamandwomens.org/medical_professionals/career/cpps/PeerSupport.aspx
National Quality Forum (Care of the Caregiver, an endorsed safe practice)
http://www.hfap.org/pdf/patient_safety.pdf
Massachusetts General Hospital (checklist for coaches supporting colleagues after AE)
http://www.mitsstools.org/uploads/3/7/7/6/3776466/mgh_checklist_adverse-event-disclosure-guidelines.pdf
Washington University Hospital in St Louis
http://www.mitsstools.org/uploads/3/7/7/6/3776466/wusm_disclosing_serious_unanticipated_adverse_events_guidelines_07_06_21_revised_08_03_18.pdf
From the UK
Royal College of Physicians,
http://www.rcplondon.ac.uk/what-we-do/patient-safety/second-victims
From Spain
Miguel Hernández University (checklist developed by the CALITÉ group),
http://calite.umh.es/segundas-victimas

A list of 48 potential interventions for health organisations and their managers in the event of a serious AE was drawn up based on current practice. The researchers then grouped, by consensus, the items on this list into mutually exclusive categories, and clearly defined each of the resulting interventions. Three did not included because they assessed similar aspects. Managers and safety coordinators of hospitals and health districts were presented with two questions about each intervention type: 1) to what degree the intervention was implemented in their organisation; and 2) to what extent they thought it was potentially useful (regardless of whether it was currently implemented in their organisation). Both questions were scored on a 5-point Likert-type scale (none/very low, low, moderate, high or very high). With the maximum possible scores for each dimension and for the entire survey being 5 and 25 points respectively. Internal consistency of the implementation subscale was 0.94 and for utility subscale 0.98. Legibility and acceptability of the questions was tested before starting the field trial. The same protocol was followed for the study in all 8 regional health services. An English translation of the questionnaire is shown in Additional file 1: Appendix I.

The questionnaire included 45 interventions proposed for second and third victims, covering: alleviating the impact of AE on second victims; peer support or specialized counselling for second victims; training in patient safety; reducing the rate of AE; open communication with patients harmed by AE and/or their relatives; civil liability cover and legal advice offered to health professionals; procedures for reporting incidents and AE; methods for analysing the causes of AE; crisis management plans; distribution of responsibilities among managerial staff; and the organisation’s approach to communication in the event of an AE, as well as specific actions with residents.

### Statistical analysis

Student’s t-tests were used for comparing the scores in the five dimensions between managers and patient safety coordinators. Using forward conditional logistic regression analysis, differences were identified in perceived implementation and usefulness of the interventions between these types of professionals and between levels of care. In both cases, intervention ratings were considered as independent variables and the dependent variable was in the first case whether the professionals were managers or safety coordinators, and in the second case, whether the organisations were hospitals or primary care centres. Some subjects did not respond to all questions. A listwise deletion approach was applied. We considered that differences were considered statistically significant when *p* < 0.05.

### Ethical approval

The study was approved by the Clinical Research Ethics Committees of the Valencia Primary Care Organisation (CEIC APCV) and the Hospital Universitario Fundación Alcorcón (CEIC HUFA). The STROBE guide was used to design the study and prepare this paper.

## Results

A total of 406 professionals completed the survey: 197 managers of hospitals and primary care centres (survey response rate of 60 %) and 209 safety coordinators (survey response rate 68 %). Responding professionals belonged to 115 hospitals (58 % of the total hospitals invited to participate), representing 32 % of all the public hospitals in these regions, and 132 primary care districts (79 % of the total primary care districts invited to participate). Participants without professionals in training did not response questions referring medical residents.

### Interventions for second victims

Overall, the degree of implementation of the proposed interventions was perceived to be greater in hospitals (mean 14.1, SD 3.5) than in primary care (mean 11.8, SD 3.1) (*p* < 0.001). On the other hand, no significant differences in implementation ratings were observed between managers and safety coordinators (mean 13.3, SD 3.0 vs mean 12.6, SD 3.7 respectively; *p* = 0.18). Considering the responses for the five dimensions in which interventions were grouped, there were differences between hospital and primary care professionals (Table 2), and between managers and patient safety coordinators (Tables 3 and 4).

**Table 2:** Implementation and usefulness of interventions to prevent the impact of adverse events on second and third victims

	Implementation			Usefulness		
Dimensions	Primary care	Hospital	P*=	Primary care	Hospital	P*=
Safety culture	2.6	3.2	0.001	3.5	3.7	ns
Crisis plan	2.5	3.1	0.001	3.6	3.8	0.05
Open communication with patients and/or relatives	2.2	2.5	0.002	3.4	3.4	ns
Support for second victims	2.3	2.5	0.022	3.6	3.6	ns
Public communication and the organisation’s reputation	2.7	2.9	0.01	3.6	3.7	ns

Comparison of the views of hospital and primary care professionals

*N* = 406

On each dimension, scores could range from 1 to 5

*from the Student’s t-test for independent samples

**Table 3:** Implementation and usefulness of interventions to prevent the impact of adverse events on second and third victims

Hospitals	Implementation			Usefulness		
Dimensions	Managers	Coordinators	P*=	Managers	Coordinators	P*=
Safety culture	3.1	3.3	ns	3.7	3.6	ns
Crisis plan	2.9	3.3	0.016	3.7	3.9	ns
Open communication with patients and/or relatives	2.2	2.8	0.001	3.4	3.5	ns
Support for second victim	2.3	2.7	0.001	3.5	3.7	ns
Public communication and the organisation’s reputation	2.8	3.1	0.024	3.7	3.8	ns

Comparison of the views of hospital managers and patient safety coordinators

*N* = 192

On each dimension, scores could range from 1 to 5

*from the Student’s t-test for independent samples

**Table 4:** Implementation and usefulness of interventions to prevent the impact of adverse events on second and third victims

Primary care	Implementation			Usefulness		
Dimensions	Managers	Coordinators	P*=	Managers	Coordinators	P*=
Safety culture	2.7	2.6	ns	3.5	3.5	ns
Crisis plan	2.4	2.7	0.035	3.6	3.5	ns
Open communication with patients and/or relatives	2.0	2.5	0.001	3.4	3.4	ns
Support for second victim	2.2	2.5	0.005	3.6	3.5	ns
Public communication and the organisation’s reputation	2.4	3.0	0.001	3.6	3.6	ns

Comparison of the views of primary care managers and patient safety coordinators

*N* = 214

On each dimension, scores could range from 1 to 5

*from the Student’s t-test for independent samples

Measures related to “Support for second victims” showed the least coverage in surveyed organisations with 71 and 61 % of the participants from hospitals and primary care respectively reporting that there was no protocol in place to treat second victims, and 70 and 66 % of the participants from hospitals and primary care respectively saying that there was no programme to guide, counsel, support and help second victims in their organization. In addition, in many cases (45 and 55 % of participants from hospitals and primary care respectively) no member of staff was assigned as a contact person for professionals involved in an AE. Regarding the dimension concerned with “Open communication with patients and/or relatives” 30–60 % of participants considered that 8 out of the 10 proposed interventions had not been implemented at all in their organisations.

### Interventions for third victims

Responses to the “Crisis management plan” dimension, yielded 35 % of hospital and 43 % of primary care professionals reporting that there was no crisis plan for serious AE in their organization, additionally in the primary care setting, no crisis committee had been set up for such AE in 34 % of cases. Regarding the “Public communication and the organisation’s reputation” dimension 30–60 % of managers and safety coordinators described that 6 out of 9 of the proposed interventions had not been implemented at all in their organisations. Additional file 2: Tables S1 and S2 list the interventions with the lowest and highest implementation ratings in hospital and primary care centres.

Notably, regarding the “Safety culture”, as many as 36 % of participants reported that studies were not being carried out to assess the rate of AE in their organization, while 36 % recognized that there was no follow-up of the effectiveness of AE preventive measures, and a quarter affirmed that no training was provided for residents covering patient safety and there was no scrutiny on the safety culture among the staff.

Table 5 lists the interventions for which the opinions of managers and coordinators differed regarding the actual degree of implementation in their organisations.

**Table 5:** Interventions to prevent the impact of adverse events on second and third victims

		95 % CI of OR	
	OR	Lower	Higher
Regular studies are carried out to assess knowledge, attitudes and behaviours related to patient safety (safety culture) among the staff (including management team).	0.2	0.1	0.6
Our reporting system is organised in such a way that it is NOT possible to identify professionals who have been involved in incidents or AE to protect their legal position.	3.2	1.6	6.3
A crisis plan has been developed that sets out what to do in the event of a serious AE in one or more patients.	0.4	0.2	0.8
We have a protocol for deciding who should tell patients (or their relatives) that an AE has occurred and what, when and how they should be told.	0.5	0.2	1.0
Patients who have suffered from serious AE (or their relatives) have an identified contact person and method of communication, in the days after the incident, to provide guidance and answer their questions.	0.4	0.2	0.9
Health professionals who have been involved in a serious AE have access to a specialized professional in their own organisation for support and as a contact person with whom to share their experience to cope with their feelings of blame, stress, and loss of confidence in their professional judgement, to reduce the impact of the AE on them as second victims.	3.6	1.4	9.4
Professionals involved with serious AE are encouraged and systematically recommended to talk to peers and other colleagues to analyse what has happened and to alleviate the pressure they feel.	0.5	0.2	0.9
We have a communication plan ensuring that, in the months after news of medical errors in the organisation, positive information about our care work is released to help to build trust in the organisation and its staff.	2.5	1.2	5.2

Data are representing differences on the level of implementation in their health organisations between managers and safety coordinators

Manager = 1, Patient safety coordinator = 0

*OR* odds ratio

### Usefulness of interventions

The perceived level of usefulness of the set of proposed interventions for second and third victims was perceived to be similar in hospitals and primary care centres (mean 18.3, SD 4.1 vs mean 17.7, SD 4.3 vs, respectively; *p* = 0.35), and among managers and safety coordinators (18.1, SD 4.4 vs 18.1, SD 4.1, respectively; *p* = 0.97). A crisis plan to guide an effective response in the event of an AE and prevent the impact of the institution as third victim was considered more useful among hospital professionals (Table 2). No other differences between levels of care (Table 2) or between managers and safety coordinators (Tables 3 and 4) were observed.

The data was further analysed in order to identify which interventions were considered the most and least useful (Additional file 2: Table S3). Assessing the rate of voluntary reports of AE on a regular basis and involving patients who have suffered AE (or their relatives) in a root cause analysis, to determine the causes and try to avoid them in the future, were both considered much more useful by safety coordinators than managers (Odds Ratio 2.2, 95 % CI 1.1–4.3; and Odds Ratio 1.9, 95 % CI 1.1–3.4, respectively). On the other hand, the development of a crisis plan to cope with serious AE in patients was considered much more useful by managers than by safety coordinators (Odds Ratio 0.3, 95 % CI 0.1–0.6).

## Discussion

In this study, we aimed to ascertain what Spanish health organisations are doing to support second and third victims as well as the extent to which extent protocols have been developed for open communication with AE victims. Considering that not in all cases the professional involved should be the person informing the patient [23]. Adequate communication management could also help second victims to cope with the professional consequences of AE. Bearing in mind the lack of existing data regarding institutions as third AE victims we sought to identify how healthcare organisations approach crisis communication and what measures and plans they have deployed. Since Denham [18] used for the first time, in 2007, the term third victim to refer to the consequences of AE on healthcare organizations, research in the field remains limited, although some evidence exists linking perceived safety and a hospital’s reputation [24].

Despite the fact that managers and patient safety coordinators in Spanish healthcare organisations perceived most of the studied interventions to prevent and alleviate the impact of AE on second and third victims were considered to be useful, the same individuals who are responsible for promoting and putting into practice such interventions, reported insufficient current implementation. Similar results have been observed in a recent study carried out in a selection of hospitals in Belgium [25], although these results suggest additional limitations in Spain. Moreover, in Spanish primary care, there is less protection for second and third victims than in hospitals. The fact that the rates and severity of AE is lower in primary care settings than in hospitals [11] does not justify this situation.

Our results indicate that reporting systems are widespread in hospitals, in line with health authority policies, however there is a lack of subsequent systematic analysis of reported AE. In the primary care setting, the findings highlight failures in both systematic AE assessment and reporting systems. In accordance with other studies [26], our results show that the issue of open communication with patients who have suffered AE remains to be addressed in both hospitals and primary care settings. According to our findings, it is often not clear who should inform patients about AE, or when or how this should happen.

Regarding third victims’ protection, it was reported that most of the organisations did not have a crisis plan in place in the event of serious AE occurring in one or more patients. Current, action plans are limited to respecting the privacy of patients and professionals involved in AE. Furthermore, provisions are not made to safeguard the legal position of professionals when investigating the causes of AE, for example, using root cause analysis [16]. Indeed, the results highlight that there are no plans or resources dedicated to caring for second victims and that related policies in healthcare organisations focus exclusively on obtaining civil liability insurance cover.

### Limitations

Only internal consistency, legibility and acceptability of the questionnaire were analysed. The sample could have been biased, as those surveyed may be more likely to respond if they are already sensitive to the issue. It was assumed that all managers would report the situation in the same way. Additionally, the response rate among hospital managers was lower than expected and we do not have data to ascertain whether the reason for not responding was a lack of interest in the study or a sense that they lacked relevant information to contribute, given limitations in their experience and/or interventions in their organisation to prevent or alleviate the impact of AE on second and third victims. It seems likely that health organisations with stronger safety cultures are more sensitised to this issue and that the staff of such organisations would be more likely to respond; if this was true, the data from this study would be an over estimate of the real state of affaires. Finally, the proposed interventions are not supported by empirical studies demonstrating their effectiveness.

Given that, at least, one in three health professionals will be involved in some type of AE, managers and safety coordinators of healthcare organisations should not only be aware of these figures, but also work towards creating a supportive work environment offering support and professional help [18, 27] beyond the legal advice provided by the organisations’ civil liability insurance. Developing and implementing interventions to prepare professionals for the occurrence of AE would help reduce their vulnerability as second victims. This could have a direct economic impact in terms of a reduced loss of work days, as well as the healthcare costs associated with clinicians losing confidence in their medical judgement after the occurrence of AE [2–4]. It would also help to reduce the rate of experienced clinicians leaving their profession in the wake of AE [2]. Aside from this, there are intangible costs in terms of damage to the reputation and public perception of healthcare organisations. Additionally, along the lines of the proposals considered in this study, healthcare organisations should propose guidelines on how to interact with patients who suffer AE in an ethical way, which reduces the likelihood that patients will sue providers and increase trust in healthcare organisations and their staff [28]. As previously recommended, all these interventions require a change in healthcare organisations’ culture [5].

Future studies should analyse whether healthcare organisations with stronger safety cultures put into practice interventions focused on supporting professionals affected by AE, analysing the effects of such events in patients and the health organisation’s prestige.

## Conclusions

This is the first study in Spain attempting to describe the current practice in many Spanish health organisations to support second victims and protect the reputation of third victims. The results show that a majority of Spanish health organizations do not have a second victim support programme or a plan to protect the reputation of institution as third victims in place in the event of a serious AE occurring in one or more patients. The results also suggest the need for many Spanish institutions to fully embrace the open disclosure practice. Healthcare organisations should propose guidelines on how to interact with patients who suffer AE in an ethical way. In primary care, there is less protection for second and third victims than in hospitals.

## Additional files

Additional file 1: Appendix I. Managers and patient safety coordinators of hospitals and primary care health districts questionnaire. (DOCX 133 kb) Additional file 2: Table S1. Interventions to support second and third victims with the lowest implementation ratings. **Table S2.** Interventions to support second and third victims with the highest implementation ratings. **Table S3.** Interventions considered the most and least useful (N = 406). (DOCX 98.3 kb)

## Abbreviations

AE: Adverse event
AVERT: The adverse event response team program
BICEPS: Treatment of stress reaction prior to combat, acronym of: brevity, immediacy, centrality, expectancy, proximity, simplicity
CEIC APCV: The Clinical Research Ethics Committees of the Valencia Primary Care Organisation
CEIC HUFA: The Clinical Research Ethics Committees of the Hospital Universitario Fundación Alcorcón
GDP: Gross domestic product
SD: Estándar desviation
STROBE: Strengthening the reporting of observational studies in epidemiology.
UK: United Kingdom
USA: United States of America
