# Supplementary material for: Interventions in health organisations to reduce the impact of adverse events in second and third victims
Source: BMC Health Serv Res. 2015 Aug 22;15:341. doi: 10.1186/s12913-015-0994-x (PMC4546284; doi:10.1186/s12913-015-0994-x)
Supplement: Additional file 1: Appendix I. — Managers and patient safety coordinators of hospitals and primary care health districts questionnaire. (DOCX 133 kb) [file 12913_2015_994_MOESM1_ESM.docx]

MANAGERS AND PATIENT SAFETY COORDINATORS OF HOSPITALS AND PRIMARY CARE HEALTH DISTRICTS QUESTIONNAIRE

| **SAFETY CULTURE** |  | | |  | | | |
| --- | --- | --- | --- | --- | --- | --- | --- |
| In our hospital/health district… | Usefulness | | | Implementation | | | |
| Regular studies are carried out to determine the rate of AEs. | POOR | FAIR | GOOD | NOT YET | POOR | FAIR | EXCELENT |
| We monitor the effectiveness of preventive measures taken in response to the findings of studies undertaken to determine the rate of AEs. | POOR | FAIR | GOOD | NOT YET | POOR | FAIR | EXCELENT |
| We periodically conduct studies about knowledge, attitudes and behaviours to describe our patient safety culture. | POOR | FAIR | GOOD | NOT YET | POOR | FAIR | EXCELENT |
| We have an annual plan for training in patient safety. | POOR | FAIR | GOOD | NOT YET | POOR | FAIR | EXCELENT |
| We offer training opportunities to the medical residents of all the specialties so that they know how to act in case of AE. | POOR | FAIR | GOOD | NOT YET | POOR | FAIR | EXCELENT |
| We are especially careful when new staff (especially in ICU, operating room, day hospital) and have a protocol to ensure that renewal personnel do not have a negative impact on safety. | POOR | FAIR | GOOD | NOT YET | POOR | FAIR | EXCELENT |
| We have a reporting system for incidents and AEs enabling us to collect useful data to minimize risks for our patients. | POOR | FAIR | GOOD | NOT YET | POOR | FAIR | EXCELENT |
| Our reporting system is organized in such a way that it is NOT possible to identify professionals who have been involved in incidents or AEs to protect their legal position. | POOR | FAIR | GOOD | NOT YET | POOR | FAIR | EXCELENT |
| We encourage staff to report incidents and AEs, handling them in a non-punitive way that is understood, agreed with and valued. | POOR | FAIR | GOOD | NOT YET | POOR | FAIR | EXCELENT |
| We carried out periodically studies to assess the report rate to introduce improvements if they are needed. | POOR | FAIR | GOOD | NOT YET | POOR | FAIR | EXCELENT |
| Our patient safety policy includes find an honest relationship with the patient who has suffered an AE. | POOR | FAIR | GOOD | NOT YET | POOR | FAIR | EXCELENT |
| The management team engaged analysing risks for safety. They have at least one session each semester reviewing several sources of information, share findings and disseminate results. | POOR | FAIR | GOOD | NOT YET | POOR | FAIR | EXCELENT |
| We celebrate clinical sessions on the occurrence of clinical errors to analyse and prevent risks in the future. Confidential measures are applied if required. | POOR | FAIR | GOOD | NOT YET | POOR | FAIR | EXCELENT |
|  | | | | | | | |
| **CRISIS PLAN** |  | | |  | | | |
| In our hospital/health district… | Usefulness | | | Implementation | | | |
| A crisis plan has been developed that sets out what to do in the event of a serious AE in one or more patients. | POOR | FAIR | GOOD | NOT YET | POOR | FAIR | EXCELENT |
| In the event of serious AEs, we organize a crisis committee composed of managers of the organizations, clinicians and other healthcare and non-healthcare staff. | POOR | FAIR | GOOD | NOT YET | POOR | FAIR | EXCELENT |
| When an EA with serious consequences for any patient occurs, we assure internal communication to avoid rumours. Our procedure assures that staff should have timely and reliable information of what has happened and how to avoid it in the future. | POOR | FAIR | GOOD | NOT YET | POOR | FAIR | EXCELENT |
| For all serious AEs, we undertake an internal investigation (root cause analysis, London protocol, etc.) to determine what occurred, when, where, and how, and what were the causes, to avoid them in the future. | POOR | FAIR | GOOD | NOT YET | POOR | FAIR | EXCELENT |
|  | | | | | | | |
| **COMUNICACIÓN Y TRANSPARENCIA CON PACIENTE Y SU FAMILIA** |  | | |  | | | |
| In our hospital/health district… | Usefulness | | | Implementation | | | |
| We have a protocol for deciding who should tell patients (or their relatives) that an AE has occurred and what, when and how they should be told. | POOR | FAIR | GOOD | NOT YET | POOR | FAIR | EXCELENT |
| It has been established what roles should be played by the directors of medical services and nursing, nurse supervisors, and the medical and nursing management in the event of an AE. | POOR | FAIR | GOOD | NOT YET | POOR | FAIR | EXCELENT |
| It has been established who will interact with and inform the patient (or their relatives) that an AE has occurred. | POOR | FAIR | GOOD | NOT YET | POOR | FAIR | EXCELENT |
| The protocol to inform patients that they have suffered an AE specifies that apologizing to the patient is important and necessary | POOR | FAIR | GOOD | NOT YET | POOR | FAIR | EXCELENT |
| Patients who have suffered from serious AEs (or their relatives) can, if they wish, access their medical record. | POOR | FAIR | GOOD | NOT YET | POOR | FAIR | EXCELENT |
| The organization offers psychological support to patients who have suffered serious AEs (or their relatives). | POOR | FAIR | GOOD | NOT YET | POOR | FAIR | EXCELENT |
| Whenever there is a serious AE, we provide swift, clear, honest and complete information to the patients (or their relatives. | POOR | FAIR | GOOD | NOT YET | POOR | FAIR | EXCELENT |
| Patients who have suffered from serious AEs (or their relatives) have an identified contact person and method of communication, in the days after the incident, to provide guidance and answer their questions. | POOR | FAIR | GOOD | NOT YET | POOR | FAIR | EXCELENT |
| We launch the necessary processes to ensure that patients (or their relatives) receive appropriate compensation for harm caused by AEs. | POOR | FAIR | GOOD | NOT YET | POOR | FAIR | EXCELENT |
| Patients or their relatives may participate, at some point in the investigation of the incident, to clarify in detail what occurred and what to do to ensure that it does not happen again. | POOR | FAIR | GOOD | NOT YET | POOR | FAIR | EXCELENT |
| We have the caution to follow up the patient who has suffered an AE for a few months to make sure that the consequences have been controlled. | POOR | FAIR | GOOD | NOT YET | POOR | FAIR | EXCELENT |
|  | | | | | | | |
| **SECOND VICTIMS SUPPORT** |  | | |  | | | |
| In our hospital/health district… | Usefulness | | | Implementation | | | |
| After serious AEs, legal and professional advice is offered from the outset under the organization’s insurance policy. | POOR | FAIR | GOOD | NOT YET | POOR | FAIR | EXCELENT |
| A protocol has been developed on how to treat second victims of AEs (colleagues in the organization) to analyse what happened and how. | POOR | FAIR | GOOD | NOT YET | POOR | FAIR | EXCELENT |
| When addressing feelings and emotions of second victims, the action plan takes into account that most AEs are due to latent errors and many are not preventable | POOR | FAIR | GOOD | NOT YET | POOR | FAIR | EXCELENT |
| Health professionals who have been involved in a serious AE have access to a specialized professional in their own organization for support and as a contact person with whom to share their experience to cope with their feelings of blame, stress, and loss of confidence in their professional judgment, to reduce the impact of the AE on them as second victims. | POOR | FAIR | GOOD | NOT YET | POOR | FAIR | EXCELENT |
| Professionals involved with serious AEs are encouraged and systematically recommended to talk to peers and other colleagues to analyse what has happened and to alleviate the pressure they feel. | POOR | FAIR | GOOD | NOT YET | POOR | FAIR | EXCELENT |
| Management is always available to talk to second victims of AEs, respecting the rights and individual circumstances of these professionals. | POOR | FAIR | GOOD | NOT YET | POOR | FAIR | EXCELENT |
| We are looking for involving second victim in the search of solutions so that the AE never occurs again. | POOR | FAIR | GOOD | NOT YET | POOR | FAIR | EXCELENT |
| We have comprehensive programme to guide, counsel, support and help second victims to cope with feelings of blame, stress, and loss of confidence in their professional judgment, to reduce the impact of the AE on them. | POOR | FAIR | GOOD | NOT YET | POOR | FAIR | EXCELENT |
|  | | | | | | | |
| **COMUNICATION AND INSTITUTIONAL IMAGE** |  | | |  | | | |
| In our hospital/health district… | Usefulness | | | Implementation | | | |
| When an AE attracts intense media coverage, we are very cautious and careful about what personal data are disclosed concerning patients and health professionals involved. | POOR | FAIR | GOOD | NOT YET | POOR | FAIR | EXCELENT |
| We do not provide information to the media on an AE without first having analysed what has happened and talked to the professionals involved. | POOR | FAIR | GOOD | NOT YET | POOR | FAIR | EXCELENT |
| The communication office issues a press release as quickly as possible, to ensure proactive disclosure and clearly explain what is known at all times regarding the AE. | POOR | FAIR | GOOD | NOT YET | POOR | FAIR | EXCELENT |
| The communication office endeavours to be in regular contact with health journalists, to ensure that they are kept well informed about what has happened, in the event of AEs with extensive media coverage. | POOR | FAIR | GOOD | NOT YET | POOR | FAIR | EXCELENT |
| We designate a spokesperson to be in charge of communication and media relations. | POOR | FAIR | GOOD | NOT YET | POOR | FAIR | EXCELENT |
| When happened an AE with severe consequences for a patient with an inevitable media component, we report what has happened to the rest of the hospital to give them real information and avoid guesses. | POOR | FAIR | GOOD | NOT YET | POOR | FAIR | EXCELENT |
| We have a communication plan ensuring that, in the months after news of medical errors in the organization, positive information about our care work is released to help to build trust in the organization and its staff. | POOR | FAIR | GOOD | NOT YET | POOR | FAIR | EXCELENT |
| Our training plan includes specific training on how to inform patients (or their relatives) that an AE has occurred. | POOR | FAIR | GOOD | NOT YET | POOR | FAIR | EXCELENT |
| Residents receive training on how to act in the event of an AE. | POOR | FAIR | GOOD | NOT YET | POOR | FAIR | EXCELENT |
