# Supplementary material for: Interventions in health organisations to reduce the impact of adverse events in second and third victims
Source: BMC Health Serv Res. 2015 Aug 22;15:341. doi: 10.1186/s12913-015-0994-x (PMC4546284; doi:10.1186/s12913-015-0994-x)
Supplement: Additional file 2: Table S1. — Interventions to support second and third victims with the lowest implementation ratings. Table S2. Interventions to support second and third victims with the highest implementation ratings. Table S3. Interventions considered the most and least useful (N = 406). (DOCX 98.3 kb) [file 12913_2015_994_MOESM2_ESM.docx]

Table S1. Interventions to support second and third victims with the lowest implementation ratings.

| **Interventions with the lowest implementation ratings** | Hospital (N=192) | |  | | Primary care (N=214) | |
| --- | --- | --- | --- | --- | --- | --- |
|  | None to Moderate |  | | None to Moderate | |  |
| We have comprehensive programme to guide, counsel, support and help second victims to cope with feelings of blame, stress, and loss of confidence in their professional judgment, to reduce the impact of the AE on them. | 70.2% |  | | 65.8% | |  |
| A protocol has been developed on how to treat second victims of AEs (colleagues in the organization) to analyse what happened and how. | 70.6% |  | | 61.4% | |  |
| The protocol to inform patients that they have suffered an AE specifies that apologizing to the patient is important and necessary. | 60.4% |  | |  | |  |
| We have a protocol for deciding who should tell patients (or their relatives) that an AE has occurred and what, when and how they should be told. | 59.0% |  | | 60.3% | |  |
| When addressing feelings and emotions of second victims, the action plan takes into account that most AEs are due to latent errors and many are not preventable. | 55.4% |  | | 49.2% | |  |
| We have a communication plan ensuring that, in the months after news of medical errors in the organization, positive information about our care work is released to help to build trust in the organization and its staff. | 45.4% |  | | 45.0% | |  |
| Health professionals who have been involved in a serious AE have access to a specialized professional in their own organization for support and as a contact person with whom to share their experience to cope with their feelings of blame, stress, and loss of confidence in their professional judgment, to reduce the impact of the AE on them as second victims. | 44.6% |  | | 54.5% | |  |
| Patients or their relatives may participate, at some point in the investigation of the incident, to clarify in detail what occurred and what to do to ensure that it does not happen again. | 44.5% |  | | 31.8% | |  |
| Our training plan includes specific training on how to inform patients (or their relatives) that an AE has occurred. | 43.2% |  | | 47.9% | |  |
| Residents receive training on how to act in the event of an AE. | 38.9% |  | | 43.6% | |  |
| It has been established what roles should be played by the directors of medical services and nursing, nurse supervisors, and the medical and nursing management in the event of an AE. | 36.1% |  | | 62.6% | |  |
| A crisis plan has been developed that sets out what to do in the event of a serious AE in one or more patients. | 34.8% |  | | 42.6% | |  |
| It has been established who will interact with and inform the patient (or their relatives) that an AE has occurred. | 34.2% |  | | 43.3% | |  |
| The organization offers psychological support to patients who have suffered serious AEs (or their relatives). |  |  | | 30.7% | |  |
| We launch the necessary processes to ensure that patients (or their relatives) receive appropriate compensation for harm caused by AEs. |  |  | | 49.5% | |  |
|  |  |  | |  | |  |
| Regular studies are carried out to determine the rate of AEs. |  |  | | 36.2% | |  |
| Patients who have suffered from serious AEs (or their relatives) have an identified contact person and method of communication, in the days after the incident, to provide guidance and answer their questions. |  |  | | 36.6% | |  |
| We monitor the effectiveness of preventive measures taken in response to the findings of studies undertaken to determine the rate of AEs. |  |  | | 35.7% | |  |
| The communication office endeavours to be in regular contact with health journalists, to ensure that they are kept well informed about what has happened, in the event of AEs with extensive media coverage. |  |  | | 35.2% | |  |
| In the event of serious AEs, we organize a crisis committee composed of managers of the organizations, clinicians and other healthcare and non-healthcare staff. |  |  | | 33.8% | |  |
| We designate a spokesperson to be in charge of communication and media relations. |  |  | | 32.0% | |  |
| Whenever there is a serious AE, we provide swift, clear, honest and complete information to the patients (or their relatives) |  |  | | 31.5% | |  |
| The communication office issues a press release as quickly as possible, to ensure proactive disclosure and clearly explain what is known at all times regarding the AE. |  |  | | 31.4% | |  |
| Professionals involved with serious AEs are encouraged and systematically recommended to talk to peers and other colleagues to analyse what has happened and to alleviate the pressure they feel. |  |  | | 31.0% | |  |

We have listed only the interventions that managers and/or safety coordinators gave very low implementation ratings.

The order of the list of interventions reflects their ratings by hospital staff.

Table S2. Interventions to support second and third victims with the highest implementation ratings

| **Interventions with the highest implementation ratings** | Hospital (N=192) | Primary care (N214) | |  |
| --- | --- | --- | --- | --- |
|  | High to very high | | High to very high | |
| Our reporting system is organized in such a way that it is NOT possible to identify professionals who have been involved in incidents or AEs to protect their legal position. | 46.8% | |  | |
| We do not provide information to the media on an AE without first having analysed what has happened and talked to the professionals involved. | 43.6% | | 33.2% | |
| We have a reporting system for incidents and AEs enabling us to collect useful data to minimize risks for our patients | 38.9% | |  | |
| Patients who have suffered from serious AEs (or their relatives) can, if they wish, access their medical record. | 36.7% | |  | |
| When an AE attracts intense media coverage, we are very cautious and careful about what personal data are disclosed concerning patients and health professionals involved | 31.4% | |  | |

We have listed only the interventions that managers and/or safety coordinators gave very high implementation ratings.

The order of the list of interventions reflects their ratings by hospital staff.

Table S3. Interventions considered the most and least useful (N = 406).

|  | None to low | Moderate | High to very high |
| --- | --- | --- | --- |
| **Interventions with the highest usefulness ratings** |  |  |  |
| When an AE attracts intense media coverage, we are very cautious and careful about what personal data are disclosed concerning patients and health professionals involved. | 4.7% | 11.3% | 84.0% |
| We do not provide information to the media on an AE without first having analysed what has happened and talked to the professionals involved. | 5.8% | 10.9% | 83.3% |
| Management are always available to talk to second victims of AEs, respecting the rights and individual circumstances of these professionals. | 6.7% | 16.8% | 76.4% |
| We have a reporting system for incidents and AEs enabling us to collect useful data to minimize risks for our patients | 12.4% | 15.7% | 72.0% |
| Our reporting system is organized in such a way that is NOT possible to identify professionals who have been involved in incidents or AEs to protect their legal position. | 12.5% | 15.9% | 71.6% |
| We encourage staff to report incidents and AEs, handling them in a non-punitive way that is understood, agreed with and valued. | 12.8% | 18.6% | 68.6% |
| For all serious AEs, we undertake an internal investigation (root cause analysis, London protocol, etc.) to determine what occurred, when, where, and how, and what were the causes, to avoid them in the future. | 13.0% | 15.2% | 71.9% |
| After serious AEs, legal and professional advice is offered from the outset under the organization’s insurance policy. | 13.1% | 13.4% | 73.3% |
|  |  |  |  |
| **Interventions with the lowest usefulness ratings** |  |  |  |
| Patients or their relatives may participate, at some point in the investigation of the incident, to clarify in detail what occurred and what to do to ensure that it does not happen again. | 30.9% | 22.2% | 47.0% |
| Our training plan includes specific training on how to inform patients (or their relatives) that an AE has occurred. | 26.3% | 16.2% | 57.5% |
| When addressing feelings and emotions of second victims, the action plan takes into account that most AEs are due to organizational errors and many are not preventable. | 25.7% | 19.7% | 54.6% |
| A protocol has been developed on how to treat second victims of AEs (colleagues in the organization) to analyse what happened and how. | 25.6% | 13.6% | 60.9% |
| We have a protocol for deciding who should tell patients (or their relatives) that an AE has occurred and what, when and how they should be told. | 25.5% | 18.5% | 56.0% |
| Health professionals who have been involved in a serious AE have access to a specialized professional in their own organization for support and as a contact person with whom to share their experience to cope with their feelings of blame, stress, and loss of confidence in their professional judgment, to reduce the impact of the AE on them as second victims. | 24.6% | 14.6% | 60.8% |
| We have a communication plan ensuring that, in the months after news of medical errors in the organization, positive information about our care work is released to help to build trust in the organization and its staff. | 22.8% | 23.1% | 54.1% |
| Patients who have suffered from serious AEs (or their relatives) can, if they wish, access their medical record. | 21.2% | 18.7% | 60.1% |
